# Supplementary material for: High-resolution discrimination of homologous and isomeric proteinogenic amino acids in nanopore sensors with ultrashort single-walled carbon nanotubes
Source: Nat Commun. 2023 May 9;14:2662. doi: 10.1038/s41467-023-38399-4 (PMC10169846; doi:10.1038/s41467-023-38399-4)
Supplement: Supplementary file 1 — Supplementary Information [file 41467_2023_38399_MOESM1_ESM.pdf]

Supplementary Information for

High-resolution discrimination of homologous and isomeric proteinogenic amino acids in nanopore sensors with ultrashort single-walled carbon nanotubes

Weichao Peng,[1,2] Shuaihu Yan,[2,3] Ke Zhou,[3] Hai-Chen Wu,[2,3*] Lei Liu,[1*] Yuliang Zhao[1,4]

Affiliations:

[1]Key Laboratory for Biomedical Effects of Nanomaterials & Nanosafety, Institute of High Energy Physics, Chinese Academy of Sciences, Beijing 100049, China.

[2]University of Chinese Academy of Sciences, Beijing 100049, China.

[3]Beijing National Laboratory for Molecular Sciences, Key Laboratory of Analytical Chemistry for Living Biosystems, Institute of Chemistry, Chinese Academy of Sciences, Beijing 100190, China.

[4]CAS Key Laboratory for Biomedical Effects of Nanomaterials & Nanosafety, National Center for Nanoscience and Technology, Beijing 100190, China.

*To whom correspondence should be addressed. Email: leiliu@ihep.ac.cn; haichenwu@iccas.ac.cn

Supplementary Figures

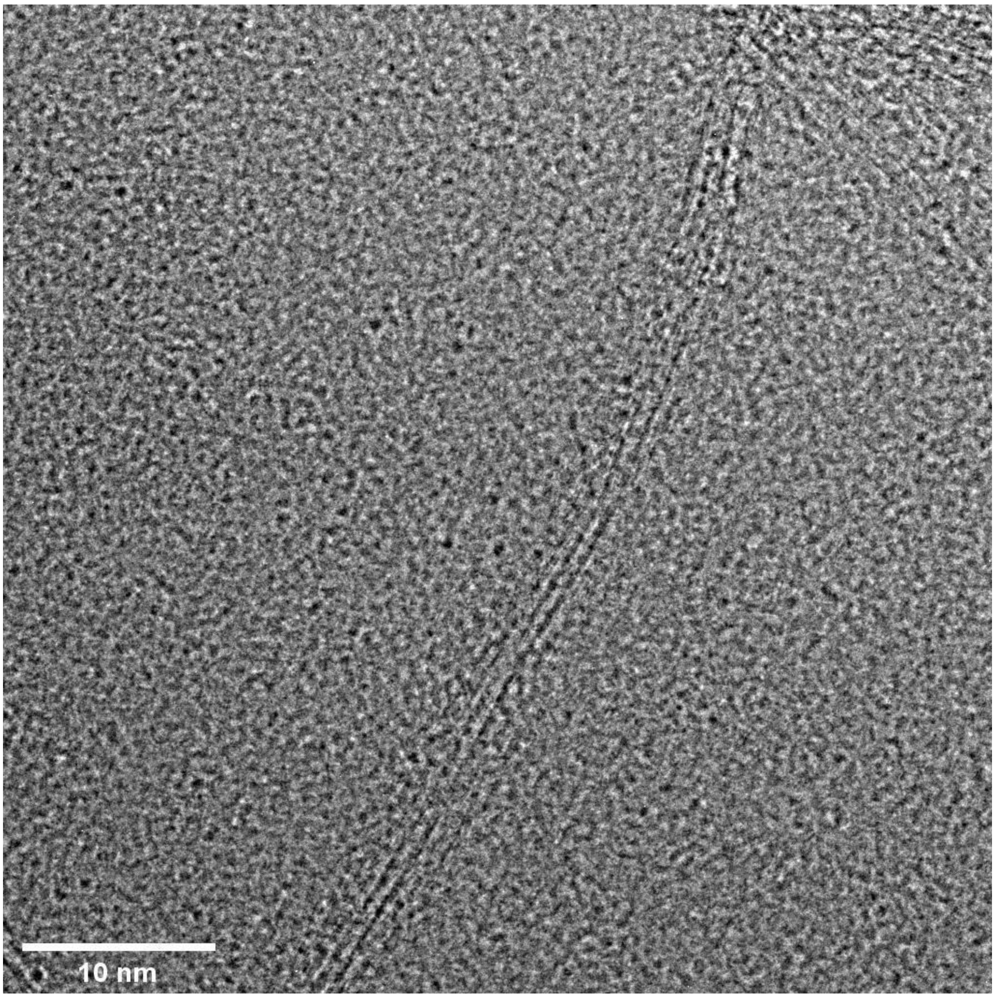

Supplementary Figure 1. A typical HRTEM image of purified SWCNTs. The HRTEM sample was prepared by sonicating purified SWCNTs for 20 min in water. The suspension was deposited onto a lacey carbon support film, and then dried in air. The imaging was carried out on a HRTEM Tecnai F20.

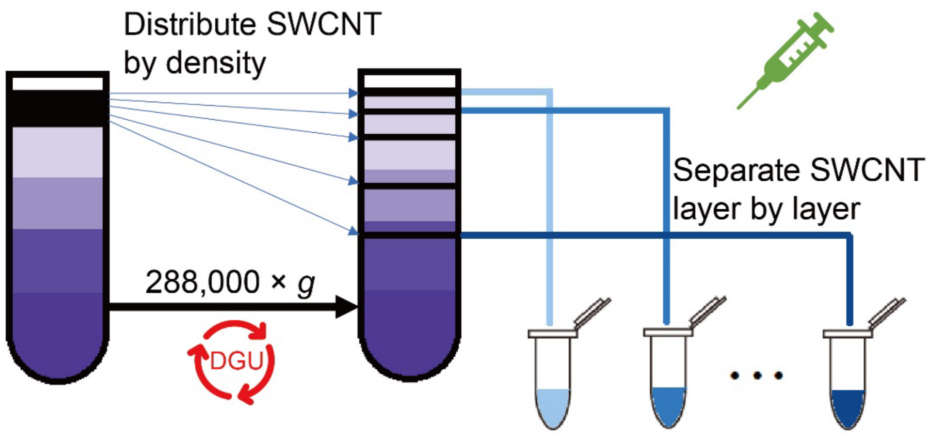

Supplementary Figure 2. Illustration of the SWCNTs separation. Density gradient ultracentrifugation (DGU) process was used to separate different SWCNTs. Step 1, the ultracentrifuge tube was filled with different concentrations of Opti Prep (iodixanol concentration from 5% to 10%) and to the top of that was added SWCNT solutions. Step 2, the DGU process was conducted under 4°C and 288,000 × *g* conditions. Step 3, the sorted SWCNT solution was fractionated layer by layer (200 μL - 400 μL) with a syringe.

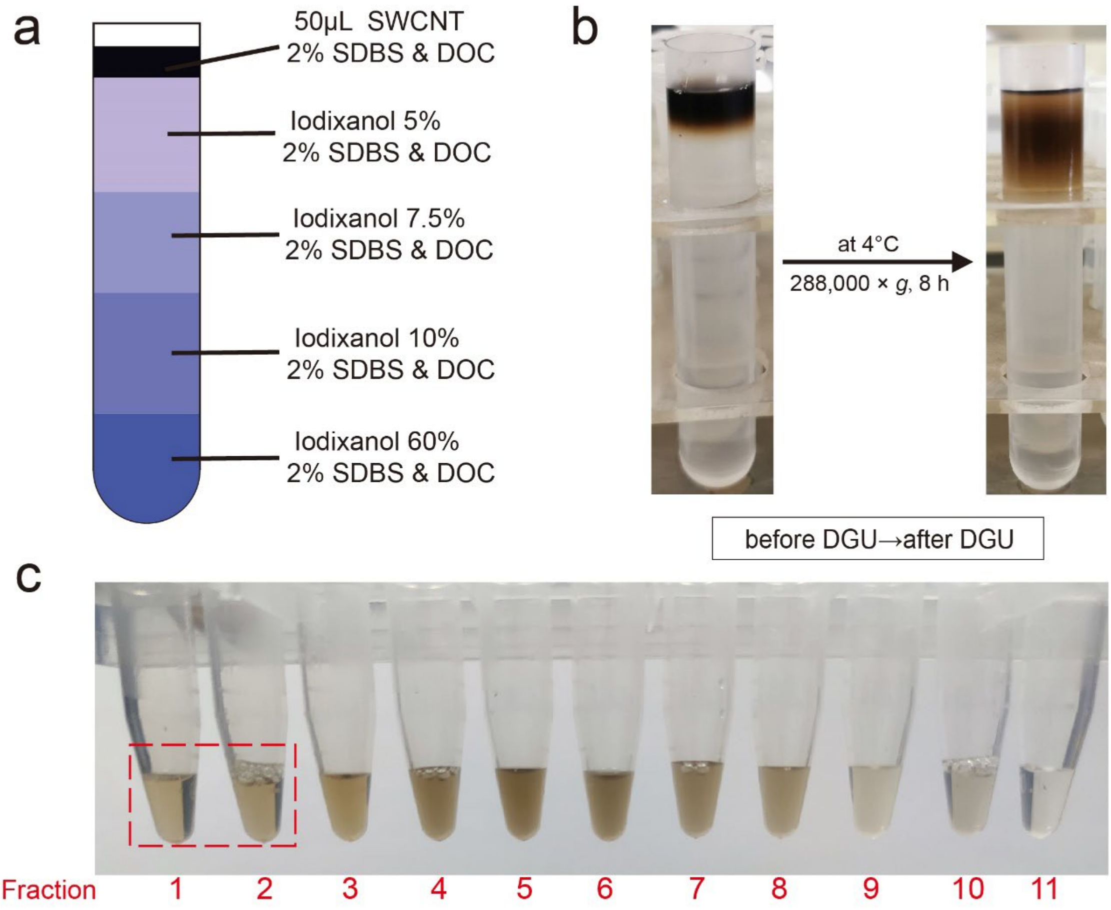

Supplementary Figure 3. Sorting SWCNTs of different length by using density gradient ultracentrifugation. (**a**) Distribution of the SWCNTs and density gradient agent solutions inside the ultracentrifuge tube. (**b**) The images of the SWCNTs before and after the DGU process. (**c**) Fractions of the layer-by-layer separation of the SWCNTs after the DGU process. The numbers indicate the layer of the fractions. The red rectangle indicates the top 2 layers of the SWCNT solution, which represents the lowest density, i.e. the shortest length and the smallest radius, of the SWCNTs. To remove the detergent from the SWCNTs mixture after the DGU process, ultrafiltration was conducted to obtain the fractions for nanopore insertion experiments.

a

b

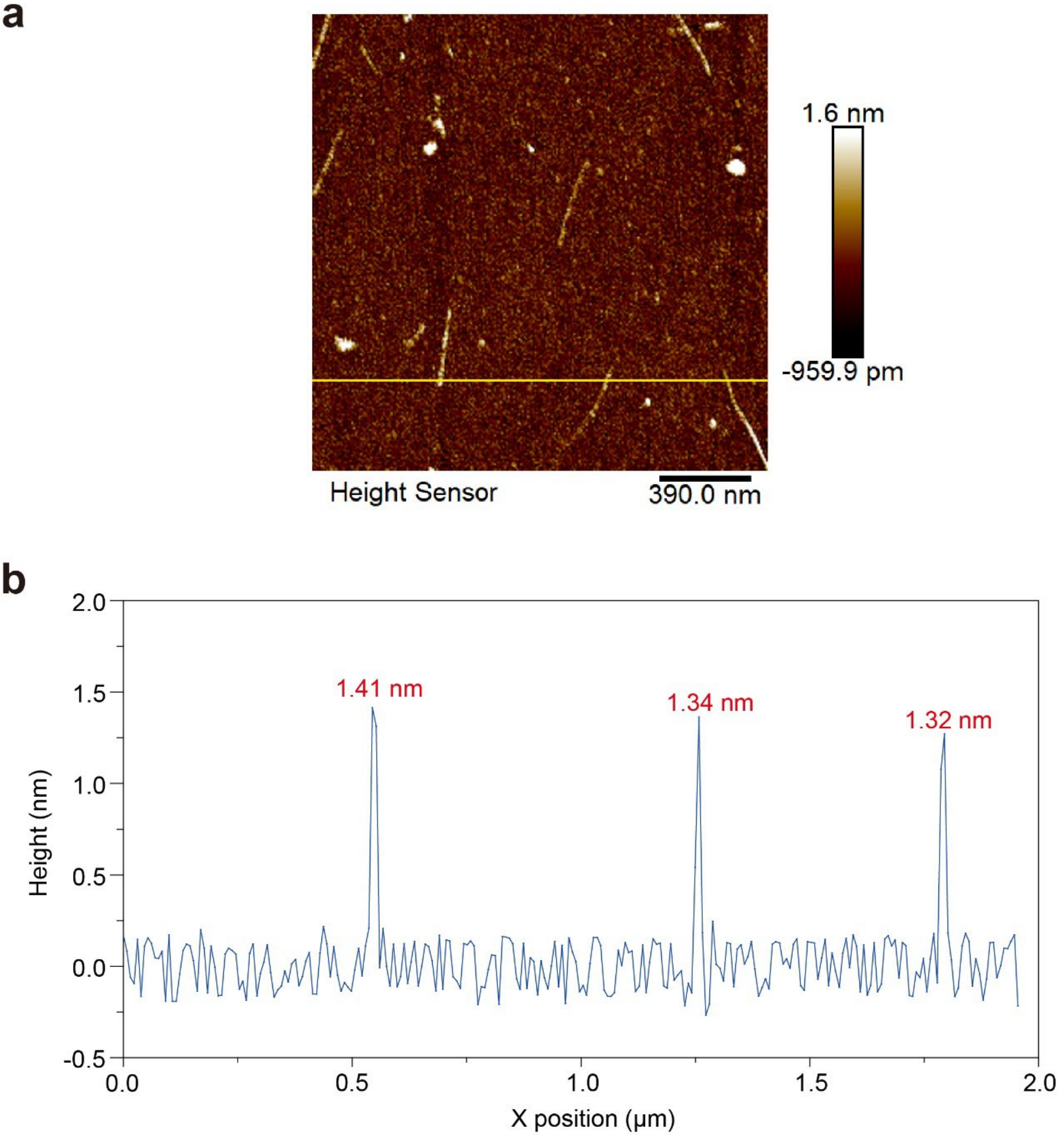

Supplementary Figure 4. AFM characterization of the long SWCNTs. (**a**) AFM image of the long SWCNTs. The nanotube length range: 200 nm to 500 nm. (**b**) Height histogram of the long SWCNTs on the yellow line in image **a**.

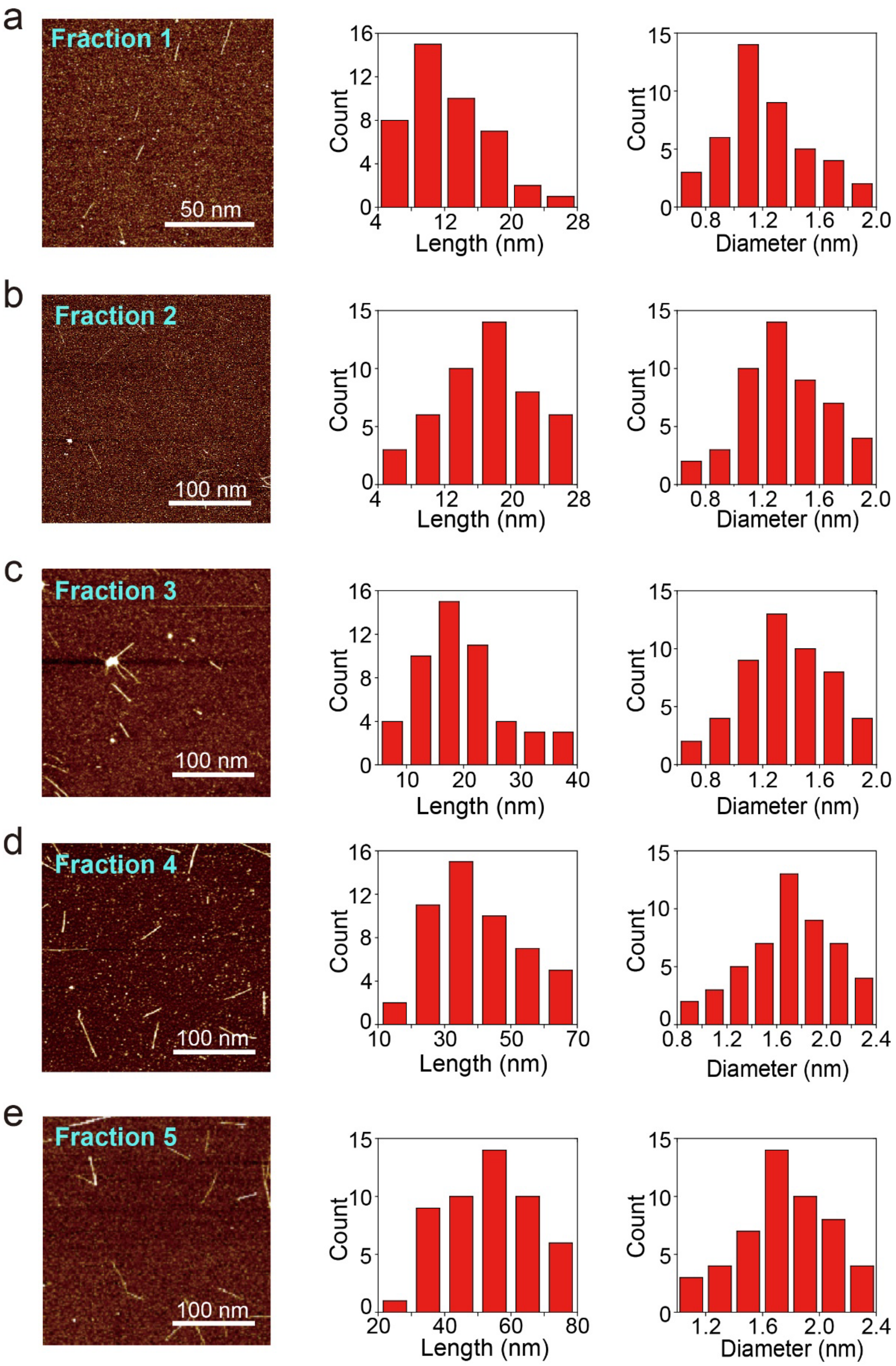
a
Fraction 1
50 nm
Count
Length (nm)
Diameter (nm)
b
Fraction 2
100 nm
c
Fraction 3
100 nm
d
Fraction 4
100 nm
e
Fraction 5
100 nm

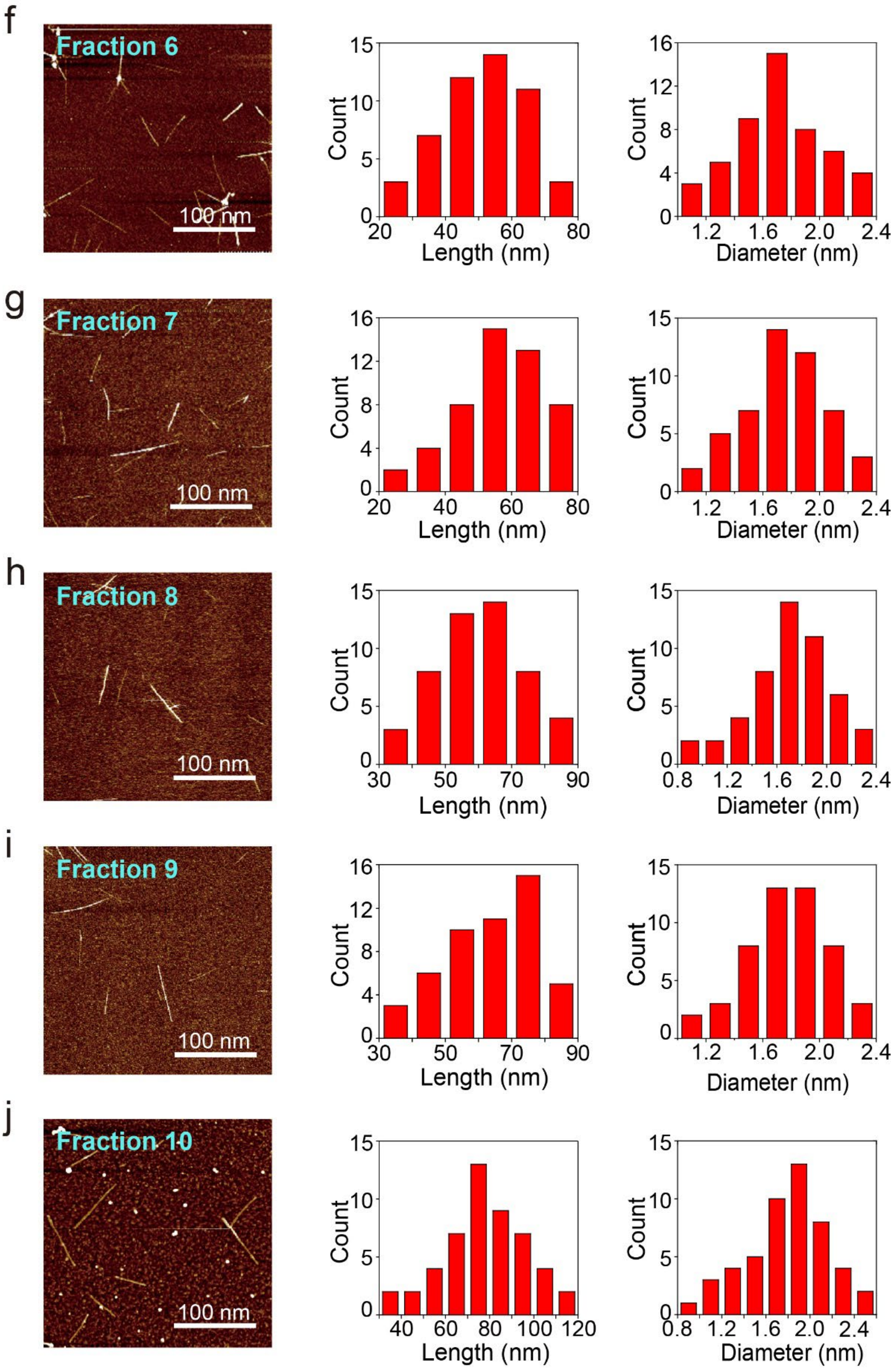

Supplementary Figure 5. AFM characterization of the ultrashort SWCNTs fractions after DGU process. The AFM image of the ultrashort SWCNTs in (**a**) fraction 1; (**b**) fraction 2; (**c**) fraction 3; (**d**) fraction 4; (**e**) fraction 5; (**f**) fraction 6; (**g**) fraction 7; (**h**) fraction 8; (**i**) fraction 9; (**j**) fraction 10. The corresponding length histograms of the samples measured from 50 tubes in each fraction are shown next to

the corresponding AFM images. The super sharp AFM tips (SuperSharpSilicon™ tips from Nano Sensors; tip radius 2.0 nm) was used to perform length measurements of the ultrashort SWCNTs.

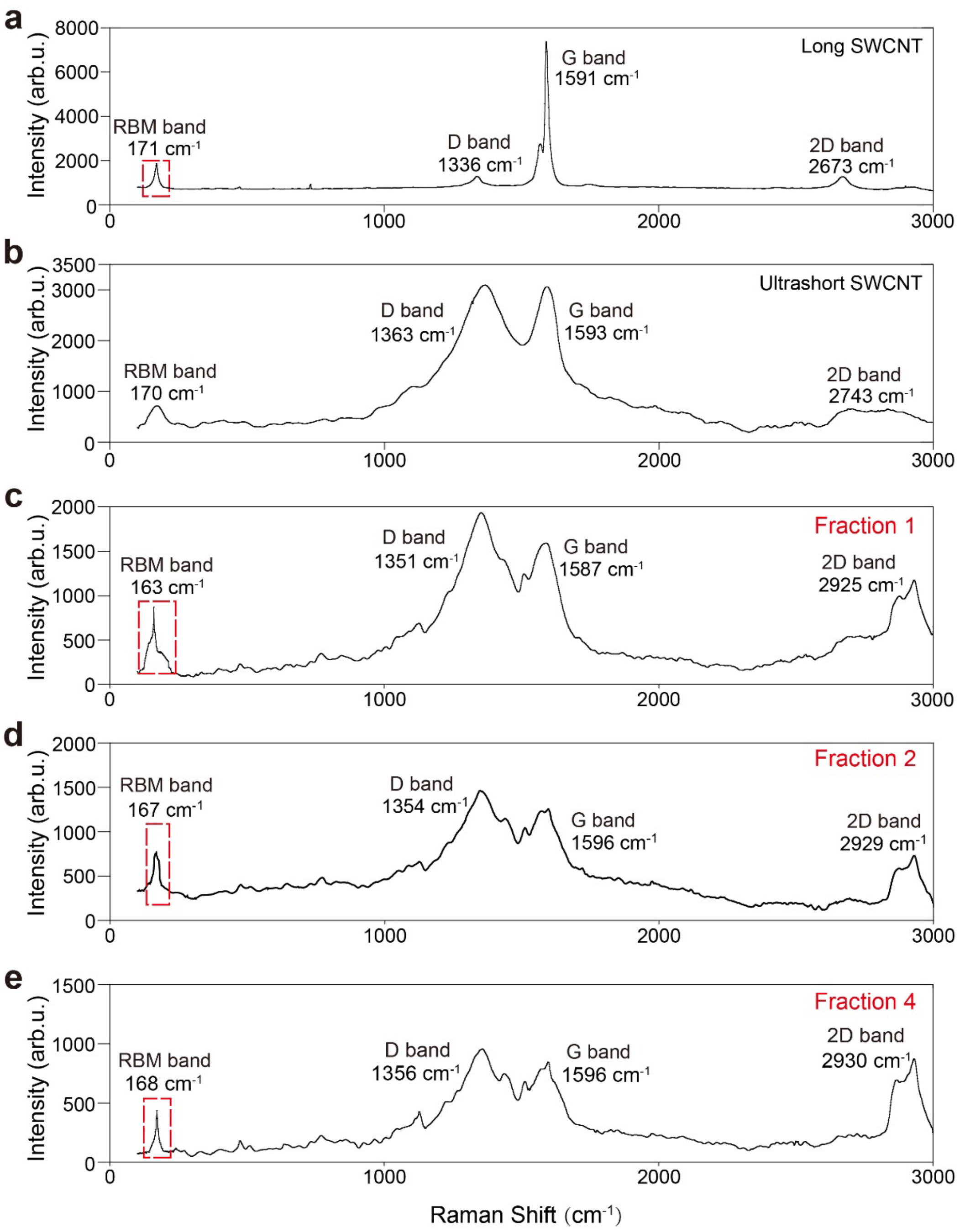

Supplementary Figure 6. Spectra data of SWCNTs by the laser Raman co-focal micro-spectrometry. (**a**) The Raman spectrum of the long SWCNTs (PureTubes™, 300 nm–4.0 μm), and radial breathing mode (RBM) band (171cm^{-1}), D band (1336cm^{-1}), G band (1591cm^{-1}) and 2D band (2673cm^{-1}) were observed. (**b**) Raman spectra of the ultrashort SWCNT (5–10 nm). (**c-e**) Raman spectra of the ultrashort SWCNT after the DGU process from fractions 1, 2 and 4. The ratio between D band and G band shows the degree of carboxylation on carbon nanotubes, which affects the water solubility of carbon nanotubes. After the DGU process, the RBM bands in fractions 1, 2 and 4 became sharpened, indicating that the SWCNTs were sorted and purified by the DGU process.

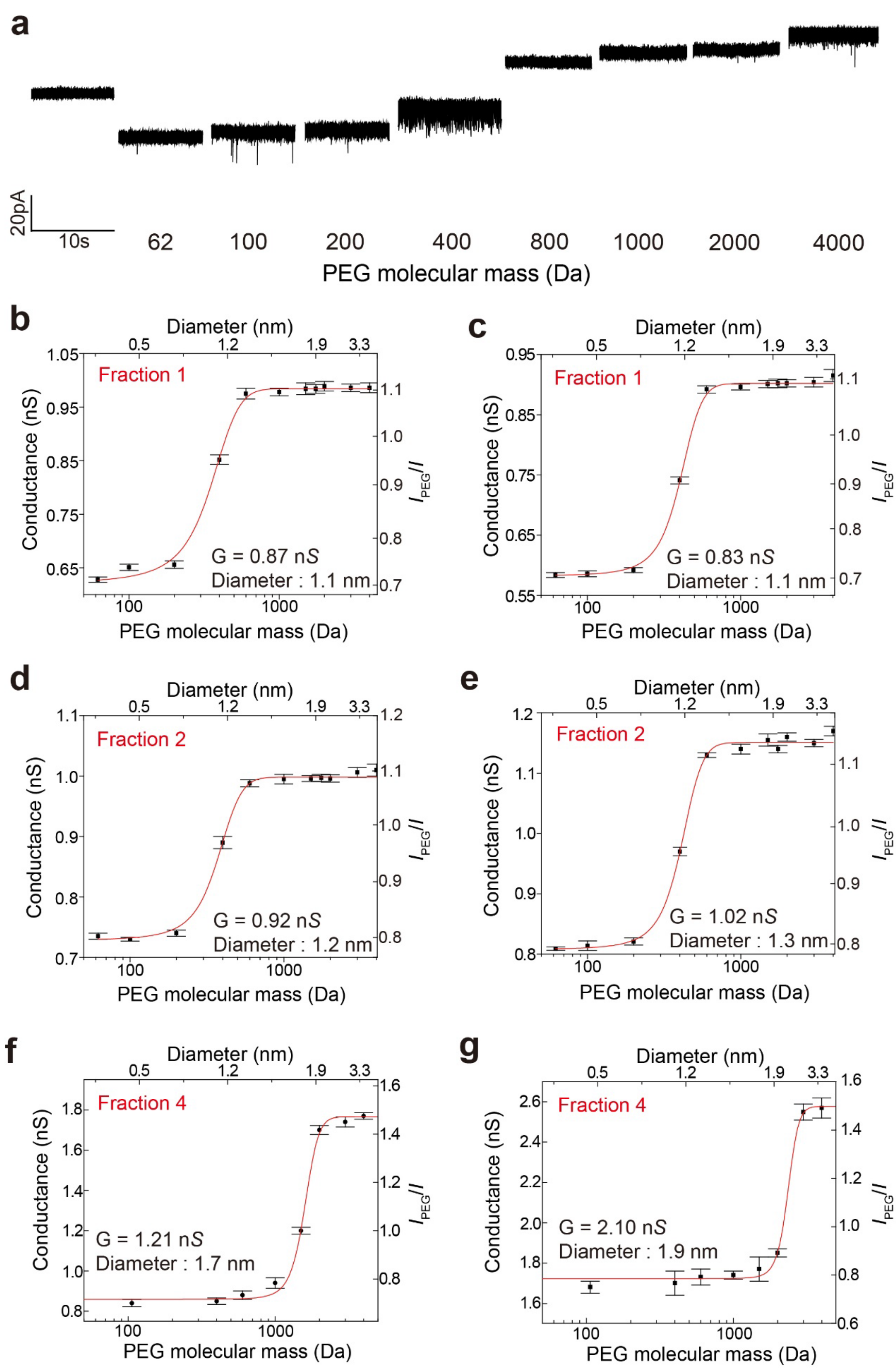

Supplementary Figure 7. Estimation of the diameters of SWCNT nanopores by threading PEG molecules of different molecular weight through the nanopore.[1] **(a)** Current traces of one SWCNT nanopore in the absence and presence of PEG molecules

with the indicated mean molecular weight (from 62 Da to 4000 Da) recorded at +40 mV in the buffer of 1.0 M KCl, 10 mM Tris, pH 8.0. (**b-g**) Curve fitting of conductance changes for the estimation of the diameter of SWCNT from fractions 1, 2 and 4. The conductance and estimated diameters are indicated in the image. Data are presented as mean values ± SD. For all the measurements, number of individual experiments n = 3.

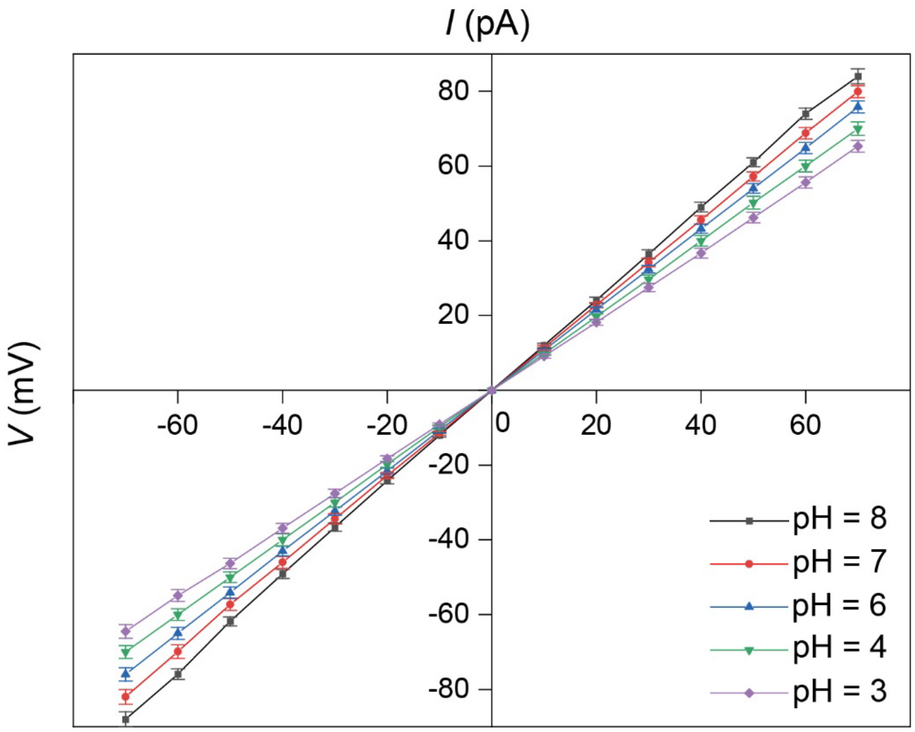

Supplementary Figure 8. Conductance changes of one SWCNT nanopore under different pH values. Experimental buffer solutions at trans and cis side were both 1.0 M KCl, 10 mM Tris, pH 8.0. The conductance of the SWCNT nanopore (from fraction 2) changed from 1.25 n*S* to 0.91 n*S*, when the pH was lowered from pH 8.0 to 3.0. The pH values were tuned by adding 1.0 M HCl solution into both the trans and cis sides. For all the measurements, number of individual experiments n = 3.

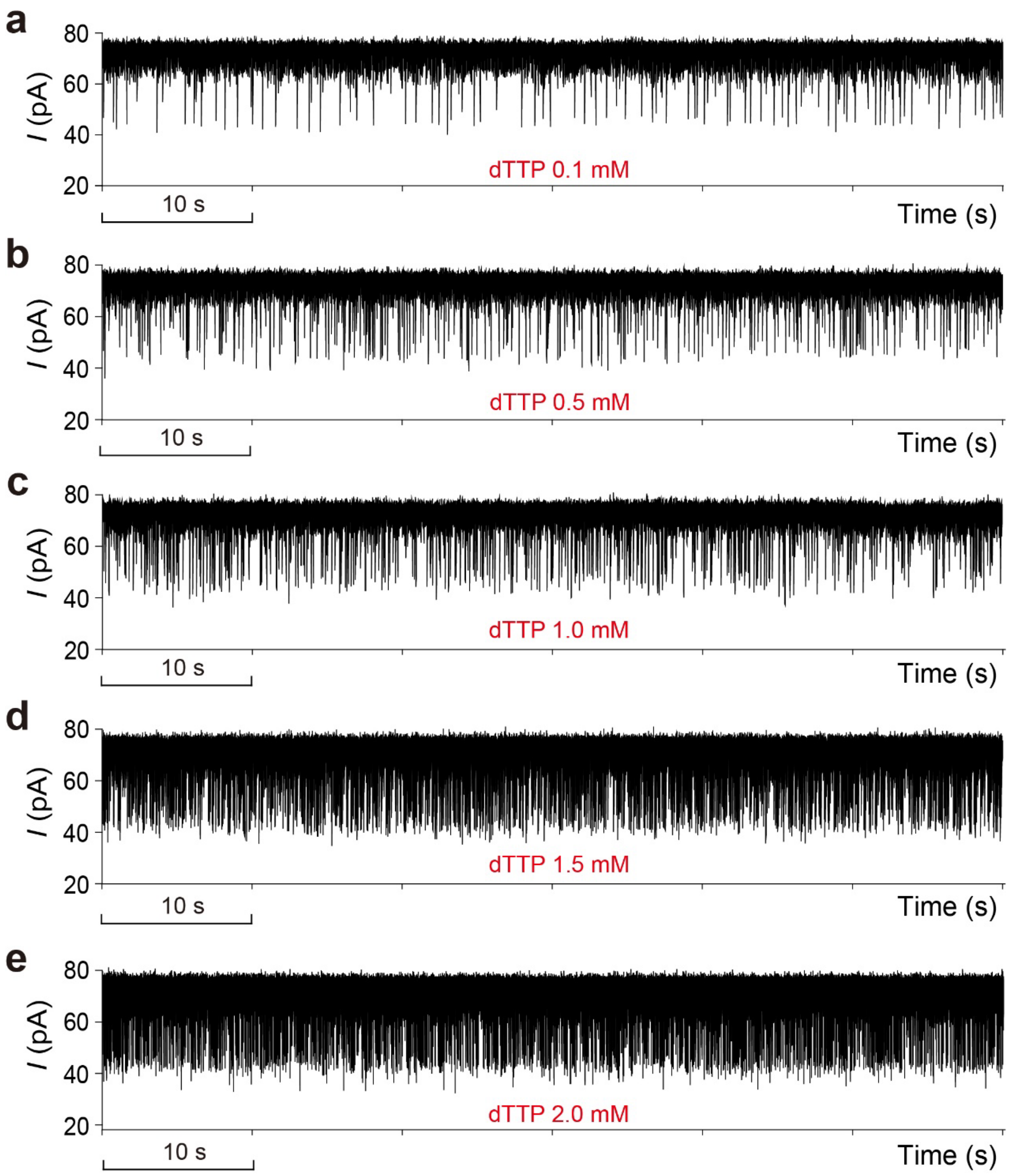

Supplementary Figure 9. Current traces generated by the translocation of different concentrations of dTTP through a SWCNT nanopore. The final concentration of dTTP is (**a**) 0.1 mM; (**b**) 0.5 mM; (**c**) 1.0 mM; (**d**) 1.5 mM**;** (**e**) 2.0 mM. All data were acquired in the buffer of 1.0 M KCl, 10 mM Tris, pH 8.0, with the transmembrane potential held at +80 mV. The conductance of carbon nanotube nanopore is 0.93 n*S* (from fraction 1). The uniformity of the current events in the presence of different concentrations of dTTP indicates that there is only one dTTP molecule in the SWCNT nanopore during the translocation process.

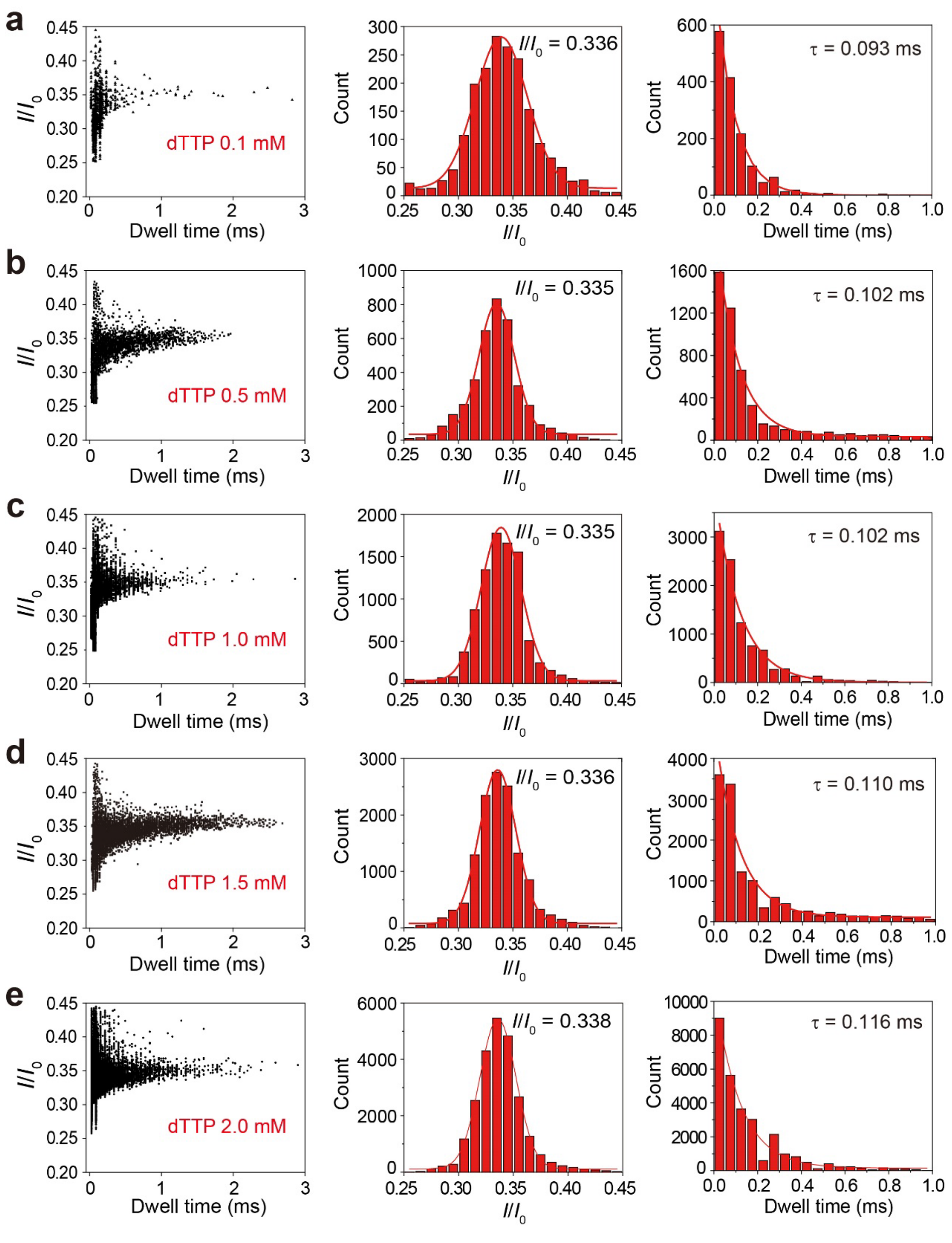

Supplementary Figure 10. Statistical analysis of the current events in Supplementary Figure 9 generated by the translocation of dTTP through a SWCNT nanopore. Scatter plots of current blockades versus event durations, histograms of the current blockades and dwell time histograms of dTTP translocation in different concentrations: (**a**) 0.1 mM; (**b**) 0.5 mM; (**c**) 1.0 mM; (**d**) 1.5 mM; (**e**) 2.0 mM. Red solid lines in the histograms of the current blockades are Gaussian fit to the histograms. Red solid lines in the dwell time histograms are single exponential fit to the histograms. Data were acquired in the buffer of 1.0 M KCl, 10 mM Tris, pH 8.0,

with the transmembrane potential held at +80 mV. The conductance of the SWCNT nanopore is 0.93 n*S* (from fraction 1).

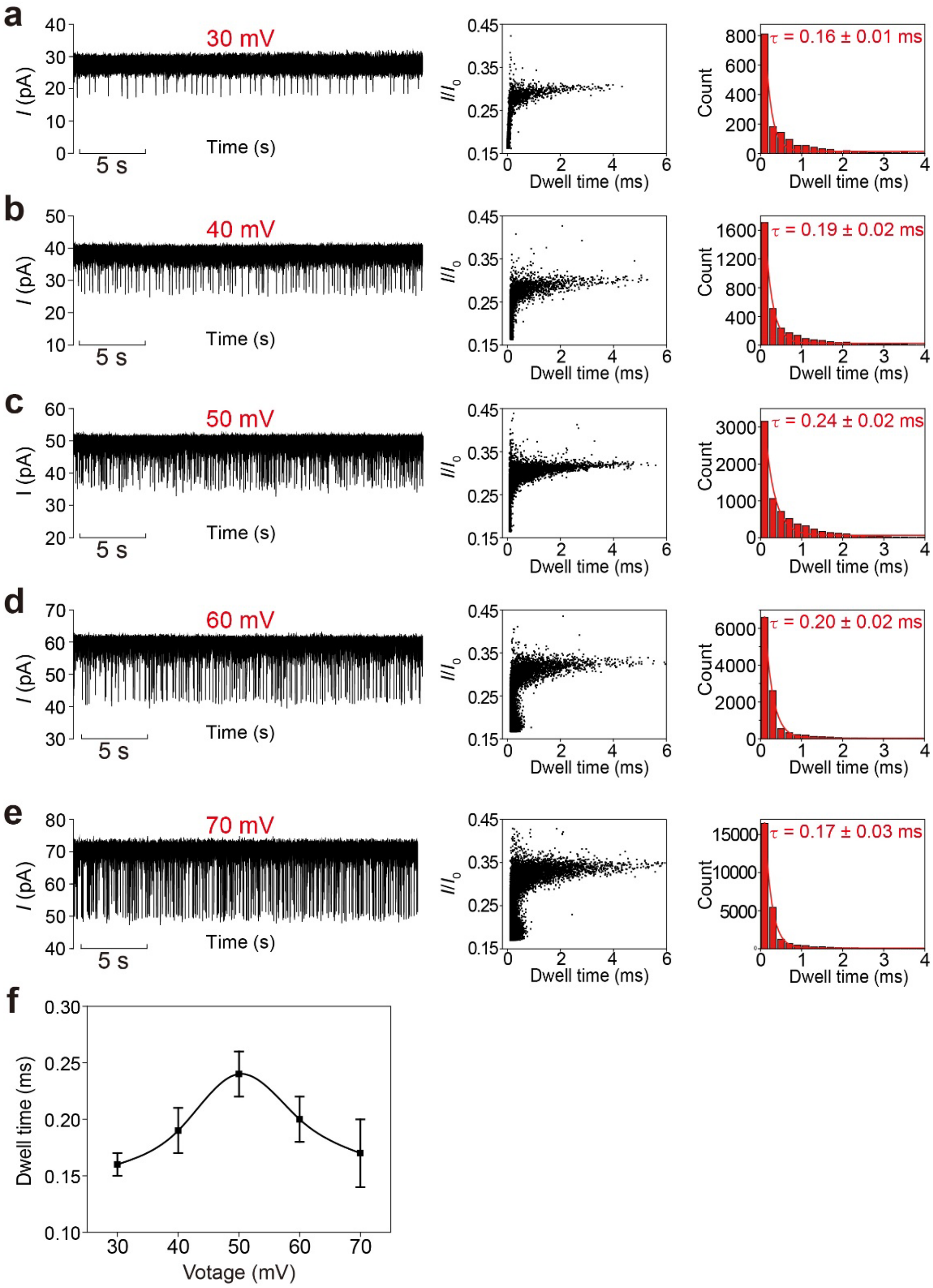

Supplementary Figure 11. Current traces and statistical analysis of the current events generated by the translocation of dTTP through a SWCNT nanopore at different voltages. Current traces (left), scatter plots of current blockades versus event durations (middle) and histograms of dwell time (right) for dTTP translocation at different voltage: (**a**) 20 mV; (**b**) 30 mV; (**c**) 40 mV; (**d**) 50 mV; (**e**) 60 mV; (**f**) 70 mV. Red solid lines in the dwell time histograms are single exponential fit to the histograms. (**f**) Curve of dwell time versus applied voltage for dTTP translocation. Data were acquired in the buffer of 1.0 M KCl, 10 mM Tris, pH 8.0 and 1.0 mM dTTP. The

conductance of the SWCNT nanopore is 0.98 n*S* (from fraction 1). Data are presented as mean values ± SD. Number of individual experiments n = 3.

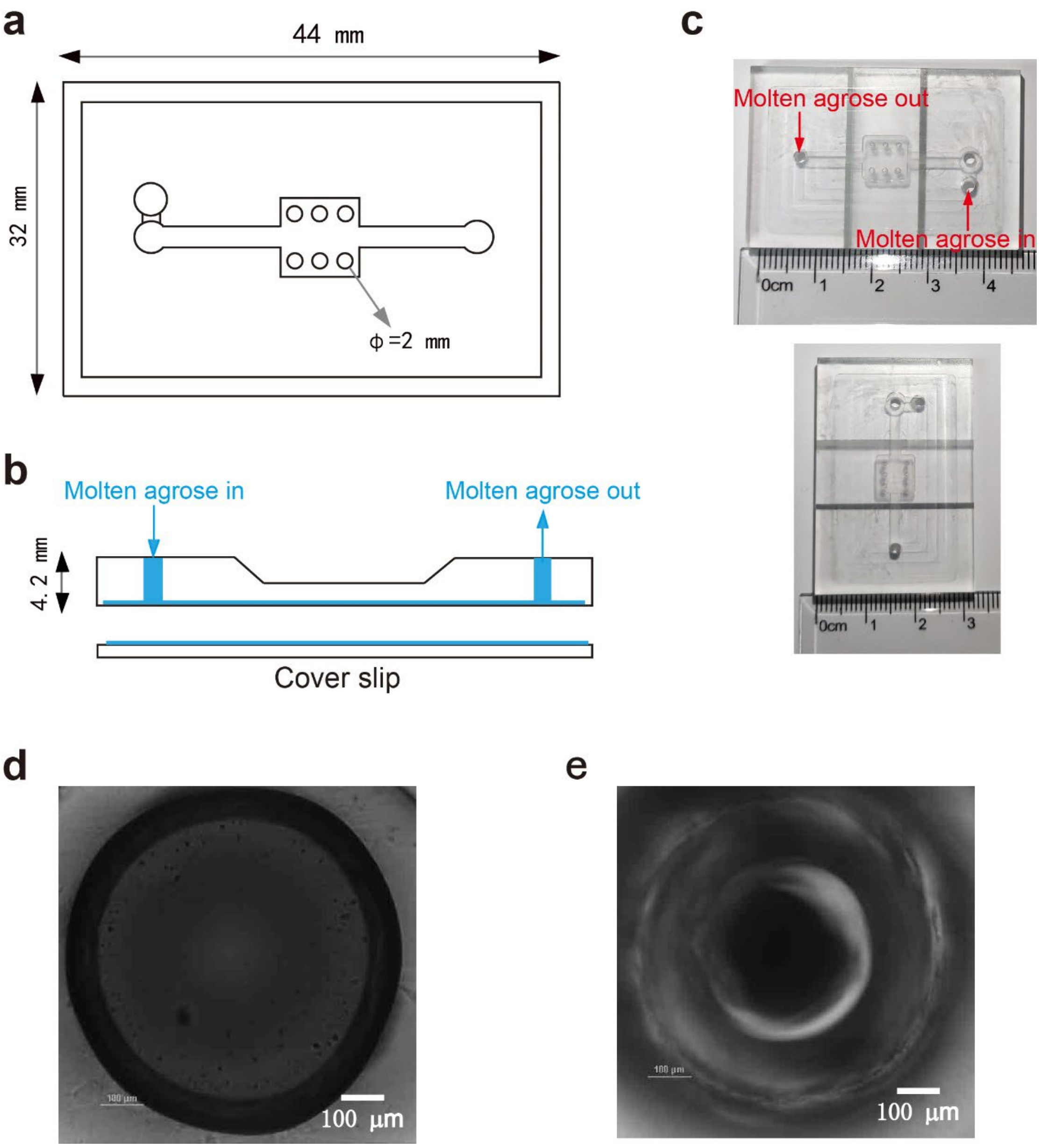

Supplementary Figure 12. The DHB measurement device and formation of lipid bilayer membrane. (**a**) Top view and (**b**) cross-sectional view of the schematic of the DHB device. (**c**) Photographs of the DHB device cell. (**d**) A Bright-field photograph of a hole of the DHB device without the droplet. (**e**) A bright-field photograph of the DHB device with the droplet, and the boundary of the droplet-hydrogel interface was the light circle in the image. When a droplet was dipped into the lipid/oil mixture (5 mg ml^{-1} DOPC in hexadecane oil) in one of the 6 holes in the center, a lipid bilayer membrane automatically formed between the droplet and the hydrogel surface. The droplet contains a solution of 1.5 M KCl, 400 μM EDTA, 30 μM Fluo-4, 10 mM Tris, pH 8.0, with one of the SWCNT, αHL or MspA nanopores. The hydrogel is composed of 3.0% (w/v) low melting temperature agarose containing a solution of 0.75 M $CaCl_2$, 10 mM Tris, pH 8.0. The droplet was controlled by a micro-manipulator to change the position on the XY-plane and the height. The experiments were performed inside a Faraday cage.

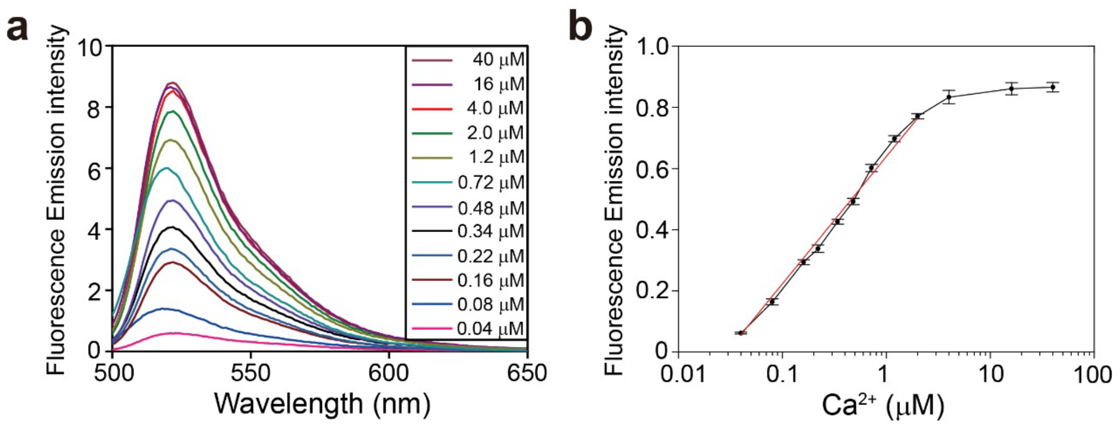

Supplementary Figure 13. Fluorescence characterization of Fluo-4, penta-potassium salt in the presence of different concentrations of Ca^{2+}. (**a**) Fluorescence emission spectra of Fluo-4 in the presence of Ca^{2+} with concentrations increasing from 0.04 to 40 μM. Excitation wavelength: 488 nm. The final concentration of Fluo-4 is 25 μM. The experiment was performed in a quartz cuvette at 298 K. (**b**) Curves showing Ca^{2+}-dependence of normalized fluorescence emission of Fluo-4. Excitation: 488 nm; emission: longpass > 520 nm. The linear range of Ca^{2+} concentration-fluorescence intensity is from 0.04 to 2.0 μM. Data are presented as mean values ± SD. Number of individual experiments n = 3.

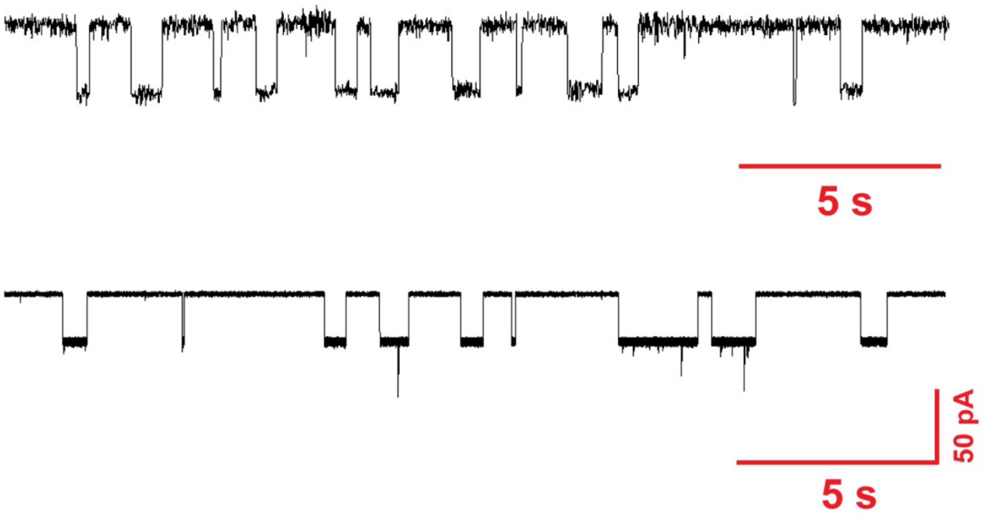

Supplementary Figure 14. Comparison of the normalized time-dependent fluorescence and the ionic current caused by stochastic binding of γCD with αHL nanopore. (**a**) An optical trace of γCD binding with αHL nanopore. A -30 mV potential was held to the DHB device to drive the Ca^{2+} ion flux through αHL, and γCD was placed in the trans side of the αHL nanopore. Fluorescent signals were monitored with a TIRF microscope (Nikon Eclipse Ti2) for 25 s. The data were filtered, background-subtracted, and normalized using NIS elements D software, ImageJ software and Python. The final concentration of γCD is 20 µM. (**b**) An ionic current trace produced by stochastic binding of γCD with αHL nanopore with the transmembrane potential held at +100 mV. The final concentration of γCD is 20 µM. All the data were acquired in the buffer of cis 0.75 M $CaCl_2$ / trans 1.5 M KCl, 10 mM Tris, pH 8.0.

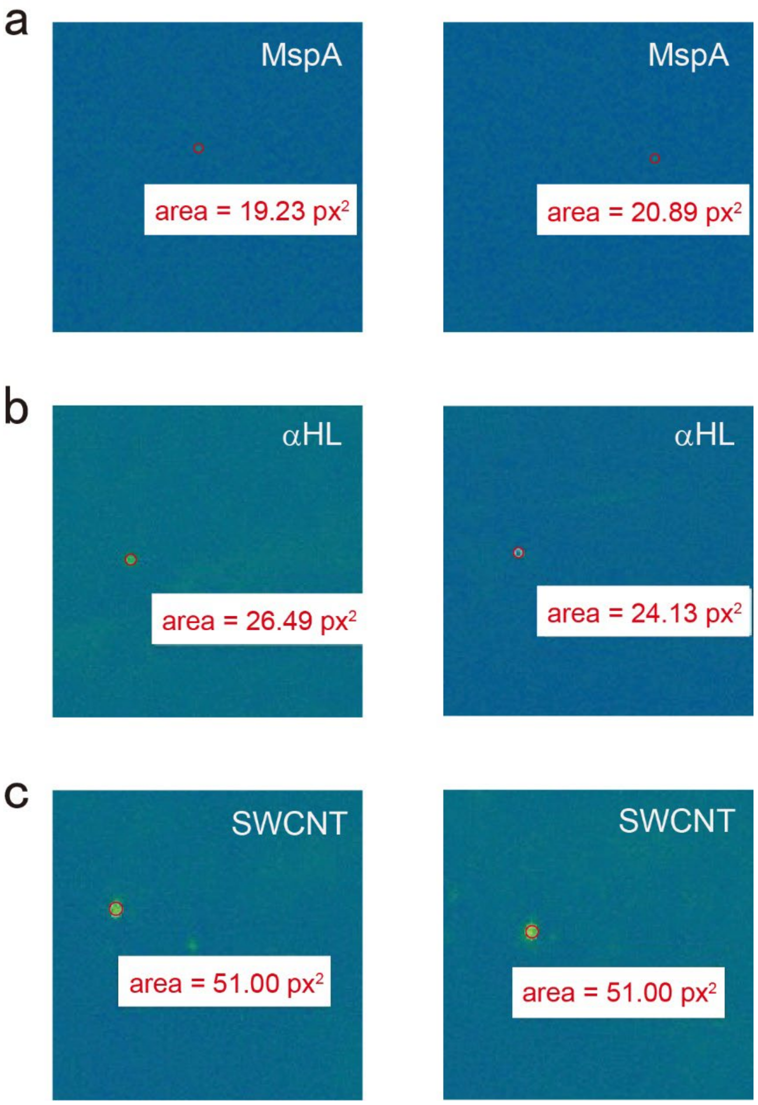

Supplementary Figure 15. Fluorescence intensity measurements of the Ca^{2+} flux through MspA/αHL/SWCNT. Fluorescent images of the DHB bilayer area containing nanopores of (**a**) MspA; (**b**) αHL; (**c**) SWCNT (from fraction 1). The data were processed with the NIS Elements D Analysis software and the details are shown in Supplementary Table 2. It is shown that the sum intensity of SWCNTs is about 2-3 times larger than that of MspA and αHL. All the fluorescence intensity data were obtained from the highest fluorescence intensity frame of the fluorescent point. The area of each image is about 10 μm × 10 μm. The droplet (trans side) contains a solution of 1.5 M KCl, 400 μM EDTA, 30 μM Fluo-4, 10 mM Tris, pH 8.0, with one of SWCNT (from fraction 1), αHL or MspA nanopores. The hydrogel (cis side) is composed of 3.0% (w/v) low melting temperature agarose with a solution of 0.75 M $CaCl_2$, 10 mM Tris, pH 8.0.

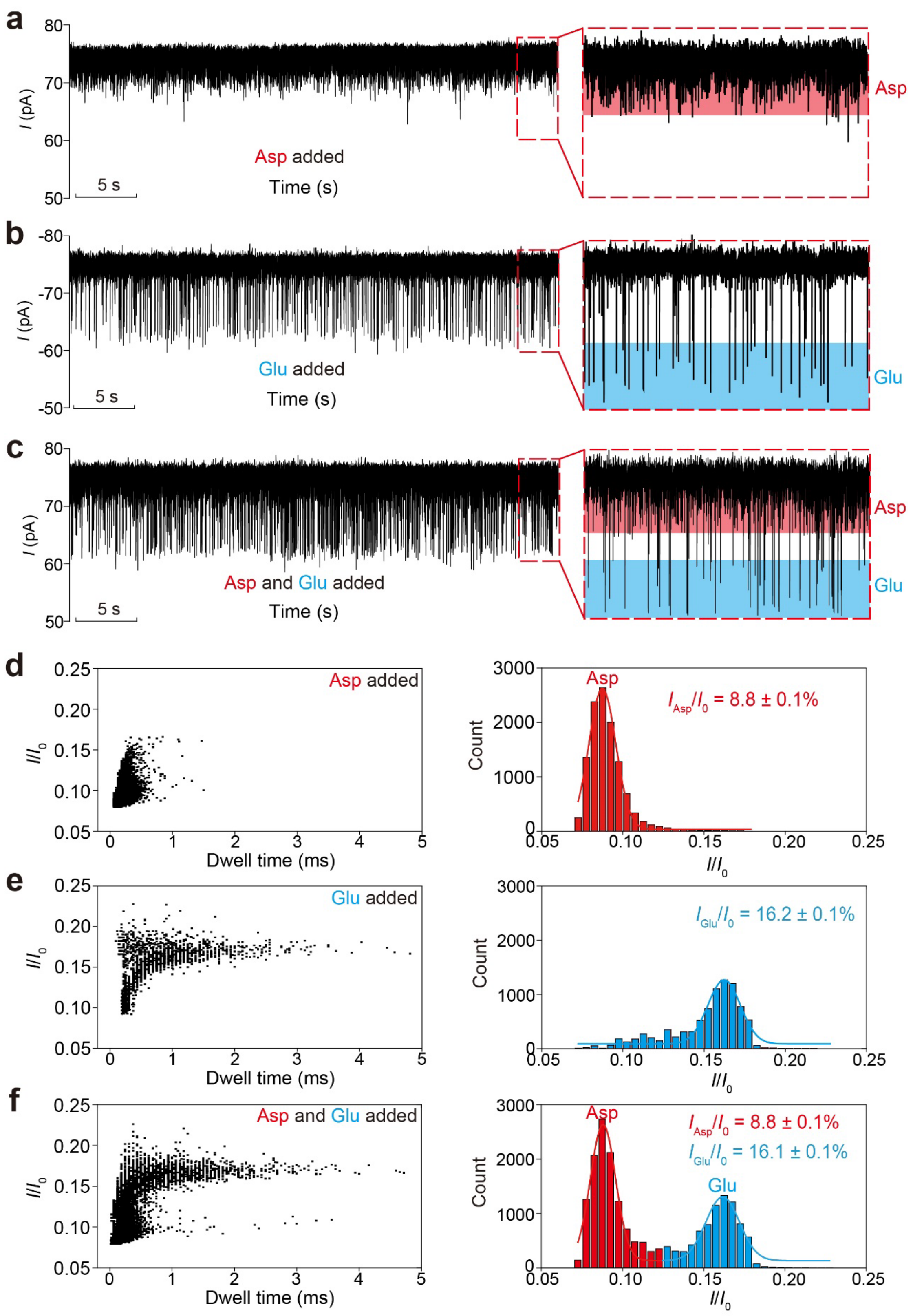

Supplementary Figure 16. Discrimination of Asp, Glu and their mixture using SWCNT nanopores. (**a**) Typical current traces of the translocation of Asp through a SWCNT nanopore at cis side. The red area represents the current events range of Asp. (**b**) Typical current traces of the translocation of Glu through a SWCNT nanopore at trans side. The blue area represents the current events of Glu. (**c**) Typical current traces

of the translocation of the mixture of Asp and Glu through a SWCNT nanopore. The red area represents the current events range of Asp and blue area represents that of Glu. (**d**) Scatter plots of current blockades versus event durations and histograms of the current blockades of the trace in **a** (Asp only at cis side). (**e**) Scatter plots of current blockades versus event durations and histograms of the current blockades of the trace in **b** (Glu only at trans side). The transmembrane potential was held at -80 mV and the current was -76 pA. (**f**) Scatter plots of current blockades versus event durations and histograms of the current blockades of the trace in **f** (mixture of Asp and Glu at cis side). The final concentration of Asp and Glu is 500 μM. Data were acquired in the buffer of 1.0 M KCl, 10 mM Tris, pH 8.0 and the transmembrane potential was held at 80 mV and the current was 76 pA unless otherwise stated. The conductance of the SWCNT nanopore is 0.95 nS (from fraction 1). Total 14 successful runs out of 17 trials.

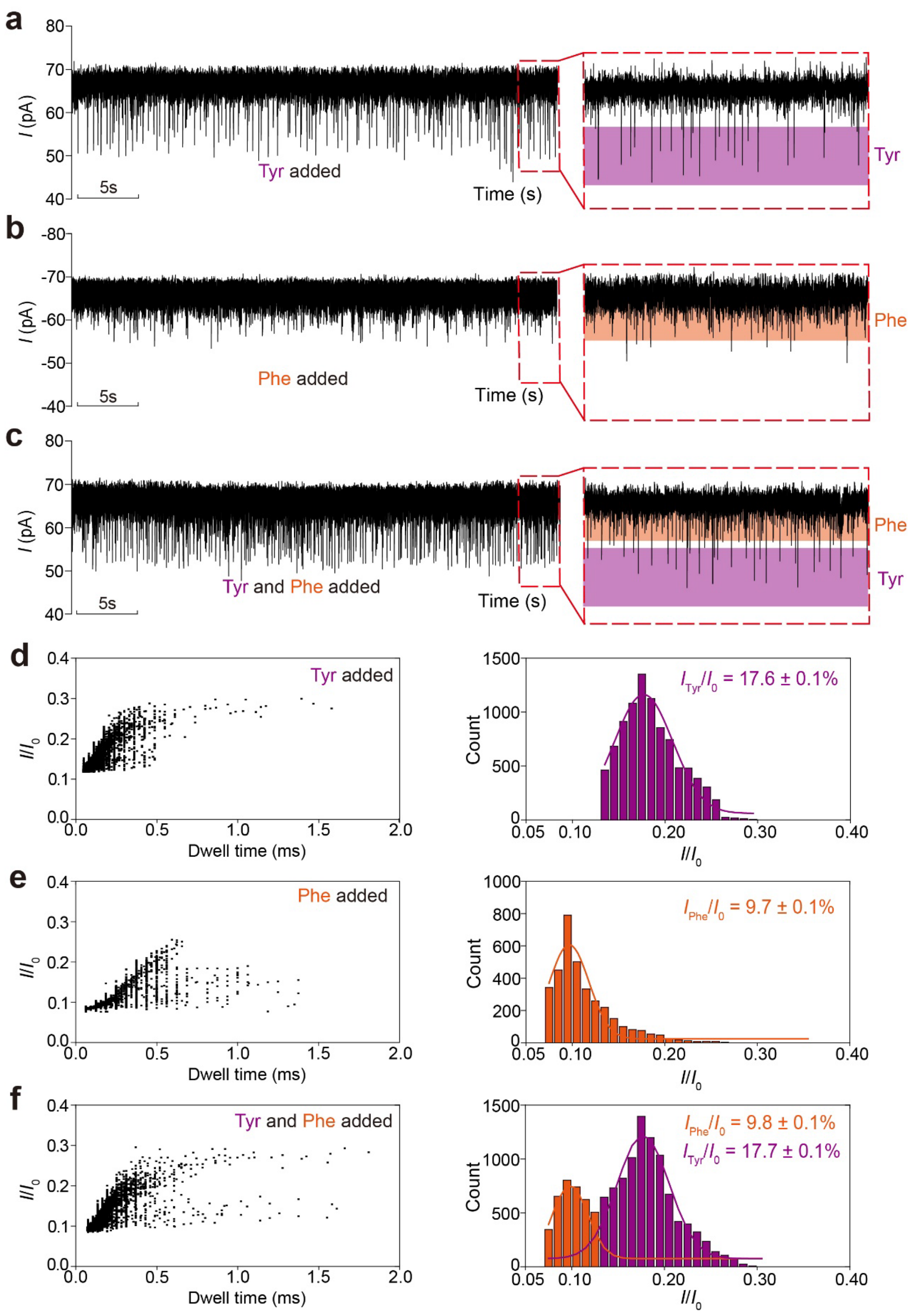

Supplementary Figure 17. Discrimination of Tyr, Phe and their mixture using SWCNT nanopores. (**a**) Typical current traces of the translocation of Tyr through a SWCNT nanopore at cis side. The orange area represents the current events range of Tyr. (**b**) Typical current traces of the translocation of Phe through a SWCNT nanopore at trans side. The purple area represents the current events of Phe. (**c**) Typical current

traces of the translocation of the mixture of Tyr and Phe through a SWCNT nanopore. The orange area represents the current events range of Tyr and purple area represents that of Phe. (**d**) Scatter plots of current blockades versus event durations and histograms of the current blockades of the trace in **a** (Tyr only at cis side). (**e**) Scatter plots of current blockades versus event durations and histograms of the current blockades of the trace in **b** (Phe only at trans side). The transmembrane potential was held at -80 mV and the current was -70 pA. (**f**) Scatter plots of current blockades versus event durations and histograms of the current blockades of the trace in **f** (mixture of Tyr and Phe at cis side). The final concentration of Tyr and Phe is 500 μM. Data were acquired in the buffer of 1.0 M KCl, 10 mM Tris, pH 8.0 and the transmembrane potential was held at 80 mV and the current was 70 pA unless otherwise stated. The conductance of the SWCNT nanopore is 0.88 nS (from fraction 1). Total 17 successful runs out of 22 trials.

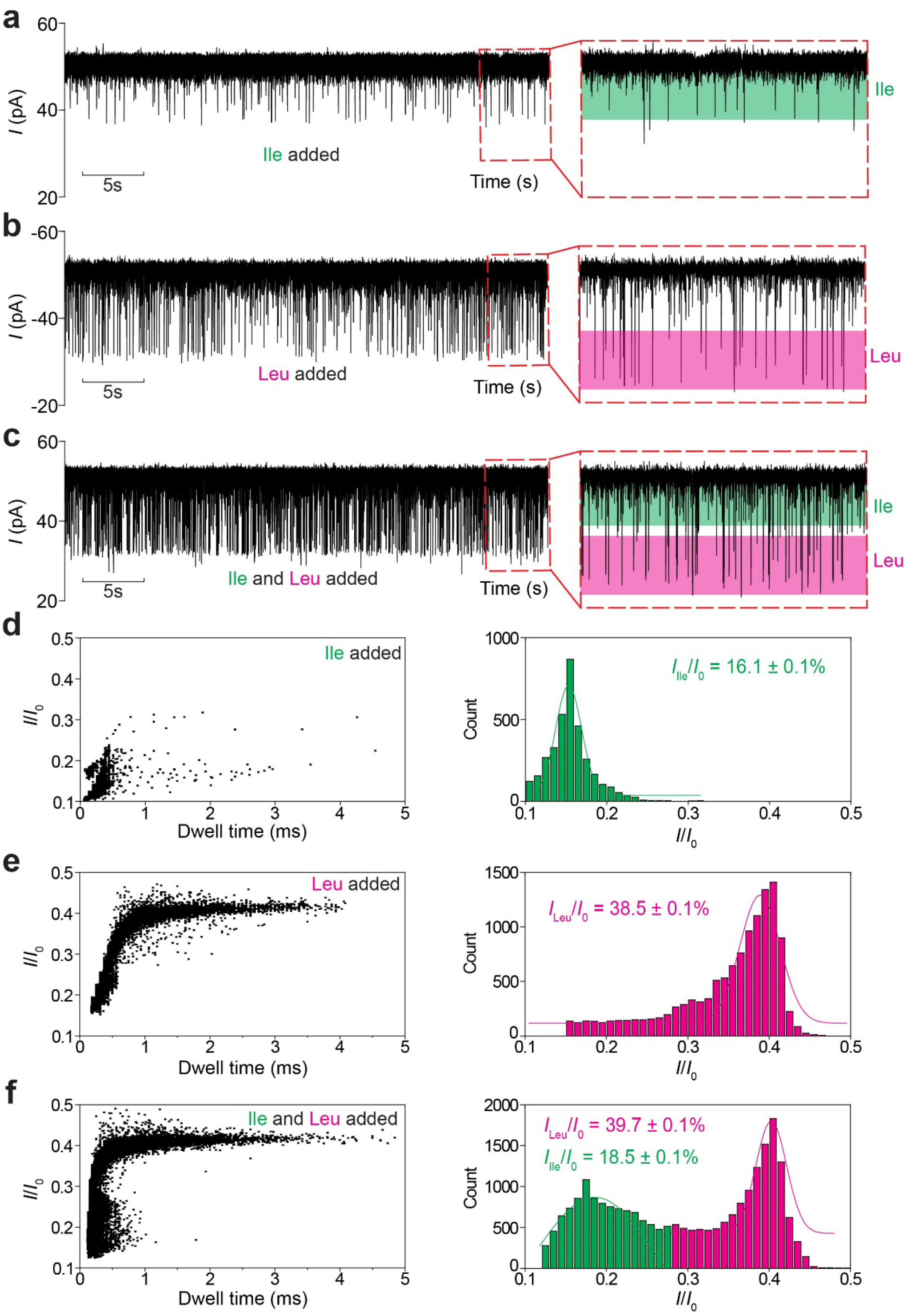

Supplementary Figure 18. Discrimination of Ile, Leu and their mixture using SWCNT nanopores. (**a**) Typical current traces of the translocation of Ile through a SWCNT nanopore at cis side. The green area represents the current events range of Ile. (**b**) Typical current traces of the translocation of Leu through a SWCNT nanopore at trans side. The pink area represents the current events of Leu. (**c**) Typical current traces

of the translocation of the mixture of Ile and Leu through a SWCNT nanopore. The green area represents the current events range of Ile and pink area represents that of Leu. (**d**) Scatter plots of current blockades versus event durations and histograms of the current blockades of the trace in **a** (Ile only at cis side). (**e**) Scatter plots of current blockades versus event durations and histograms of the current blockades of the trace in **b** (Leu only at trans side). The transmembrane potential was held at -70 mV and the current was -58 pA. (**f**) Scatter plots of current blockades versus event durations and histograms of the current blockades of the trace in **f** (mixture of Ile and Leu at cis side). The final concentration of Ile and Leu is 500 μM. Data were acquired in the buffer of 1.0 M KCl, 10 mM Tris, pH 8.0 and the transmembrane potential was held at 70 mV and the current was 58 pA unless otherwise stated. The conductance of the SWCNT nanopore is 0.83 nS (from fraction 1). Total 12 successful runs out of 20 trials.

Supplementary Table 1. Reversal potential and selectivity ratio values for three different SWCNT nanopores in Fig. 1e. All the experiments were performed in the buffer of trans 1.0 M KCl / cis 0.1 M KCl, 10 mM Tris at pH 8.0.

**Tube**	**pH**	**Reversal Potential (mV)**	**Selectivity Ratio (K^+/Cl^-)**
Tube 1	8.0	-18.0	2.14
	7.0	-16.0	1.94
	6.0	-14.0	1.78
	4.0	-9.0	1.43
	3.0	-5.0	1.18
Tube 2	8.0	-15.0	1.85
	7.0	-13.0	1.70
	6.0	-10.0	1.50
	4.0	-5.0	1.22
	3.0	-3.0	1.12
Tube 3	8.0	-10.0	1.50
	7.0	-8.0	1.38
	6.0	-7.0	1.33
	4.0	-4.0	1.17
	3.0	-2.0	1.04

Supplementary Table 2. Details of fluorescence intensity measurement in Supplementary Fig. 11.

Nanopore	Area / pixel^2	Equivalent diameter / pixel	Mean Intensity	Sum Intensity
**MspA**	19.23	4.95	88.13	881.33
	20.89	5.16	84.43	844.33
**αHL**	26.49	5.81	92.47	924.67
	24.13	5.54	88.63	886.33
**SWCNT**	51.00	8.06	100.99	3231.67
	51.00	8.06	113.96	3646.45

Supplementary Reference

1. Burns, J. R., Seifert, A., Fertig, N. & Howorka, S. A biomimetic DNA-based channel for the ligand-controlled transport of charged molecular cargo across a biological membrane. *Nat. Nanotechnol.* **11**, 152-156 (2016).
