# Supplementary material for: High-resolution discrimination of homologous and isomeric proteinogenic amino acids in nanopore sensors with ultrashort single-walled carbon nanotubes
Source: Nat Commun. 2023 May 9;14:2662. doi: 10.1038/s41467-023-38399-4 (PMC10169846; doi:10.1038/s41467-023-38399-4)
Supplement: Supplementary file 3 — Description of Additional Supplementary Files [file 41467_2023_38399_MOESM3_ESM.pdf]

File Name: Supplementary Movie 1

Description: Blinking of the Ca^{2+}-Fluo-4 fluorescent signals due to the blockage of Ca^{2+} ions through SWCNT nanopore by the presence of dTTP.

File Name: Supplementary Movie 2

Description: Blinking of the Ca^{2+}-Fluo-4 fluorescent signals due to the blockage of Ca^{2+} ions through MspA nanopore by the presence of dTTP.

File Name: Supplementary Movie 3

Description: Blinking of the Ca^{2+}-Fluo-4 fluorescent signals due to the blockage of Ca^{2+} ions through αHL nanopore by the presence of dTTP.
